# I-FABP, citrulline and non-invasive liver dysfunction indices in patients with depression – cross-sectional study results

**DOI:** 10.1186/s12876-025-04559-7

**Published:** 2026-01-03

**Authors:** Jakub Rogalski, Joanna Grzelczyk, Aleksandra Margulska, Grzegorz Mirocha, Grażyna Budryn, Dominik Strzelecki, Oliwia Gawlik-Kotelnicka

**Affiliations:** 1 Department of Old Age Psychiatry and Psychotic Disorders, Medical University of Lodz, Czechosłowacka 8/10, Lodz, 92-216 Poland; 2 Institute of Food Technology and Analysis, Faculty of Biotechnology and Food Sciences, Lodz University of Technology, Lodz, 90-537 Poland; 3 Department of Child and Adolescent Psychiatry, Medical University of Lodz, Czechosłowacka 8/10, Lodz, 92-216 Poland; 4 Department of Biostatistics and Translational Medicine, Medical University of Lodz, Lodz, 92-215 Poland; 5 Department of Affective and Psychotic Disorders, Medical University of Lodz, Czechosłowacka 8/10, Lodz, 92-216 Poland

**Keywords:** Cardiovascular risk, Citrulline, Depressive disorders, Hepatic steatosis, Intestinal fatty acid-binding protein, Intestine dysfunction, Intestine permeability, Liver fibrosis, Metabolic syndrome

## Abstract

**Introduction:**

Dysfunction of the intestinal epithelial barrier, often observed in individuals with depression and Metabolic Syndrome (MetS), may contribute to liver dysfunction and increased cardiovascular risk (CVR). However, hepatic alterations influenced by enteric dysfunction in individuals with depression remain insufficiently investigated. The aim of the study is to investigate the associations between the non-invasive indices of hepatic steatosis and fibrosis and intestinal dysfunction biomarkers in patients with depressive disorders, taking into account the presence of MetS or its components, as well as dietary, behavioral and psychosocial factors, and antidepressant intake status.

**Materials and methods:**

In this cross-sectional study, data from 116 subjects were analyzed. The intestinal function blood biomarker citrulline (CIT) and gut permeability indicator Intestinal Fatty-Acid Binding Protein (I-FABP) were assessed alongside non-invasive liver-related parameters. Metabolic, dietary, and psychometric parameters were also evaluated.

**Results:**

Median I-FABP and I-FABP/CIT ratio values were higher in individuals with Fibrosis-4 (FIB-4) Index > 1.3 than in those with FIB-4 < 1.3 (*p* = 0.027 and r_rb_=0.447; *p* = 0.018 and r_rb_=0.479, respectively). In a logistic regression model with FIB-4 > 1.3 as the dependent variable, vegetable and seed consumption, CIT levels, waist circumference (WC) and quality of life were significant predictors, with WC being the most crucial (*p* = 0.019, OR = 1.1; 95% CI: 1.017-1.205). Additionally, subjects with Hepatic Steatosis Index (HSI) > 36.0 who did not have concomitant MetS had higher CIT levels compared to those meeting both criteria (*p* = 0.016, r_rb_=0.529).

**Conclusions:**

The association between FIB-4 and I-FABP seems to reflect an increased risk of hepatic abnormalities and related CVR in individuals with depression, particularly those with suspected intestinal permeability disturbances, with CIT serving as a significant predictor.. Reduced CIT levels in participants with MetS and liver dysfunction suggest greater impairment of gut integrity than in liver dysfunction alone. Conversely, individuals with liver dysfunction, in the absence of MetS, may have preserved intestinal function, as indicated by higher CIT concentrations.

**Trial registration:**

Trial was registered with the ClinicalTrials.gov registry (ClinicalTrials.gov ID: NCT04756544, https://clinicaltrials.gov/study/NCT04756544, registration date: 11/02/2021).

**Supplementary Information:**

The online version contains supplementary material available at 10.1186/s12876-025-04559-7.

## Introduction

### An overview

 Depressive (DD) and metabolic disorders are two categories of most debilitating civilization diseases worldwide, with increasing incidences every year [1, 2]. Although the etiopathogenesis of both states is complex, in recent times, particular attention has been paid to the gut-liver-brain axis (GLBA) disruptions as possible cause of the simultaneous co-existence of neuropsychiatric and metabolic disorders, as well as various metabolic alterations separately [3–7]. GLBA refers to the dynamic, multidirectional communication network involving the intestinal microbiome, gut epithelium, liver, and brain, which is coordinated through a variety of endocrine, humoral, metabolic, and immune signaling pathways [8, 9]. Within this framework, particular attention has been paid to the dysfunction of the intestinal epithelial barrier, which is frequently observed among patients diagnosed with both depression and Metabolic Syndrome (MetS) components. Such alteration may contribute to liver function abnormalities, including the increased risk of hepatic steatosis and fibrosis. A growing body of evidence supports this hypothesis, indicating that the translocation of bacteria and toxic substances from the intestines to the portal circulation may trigger chronic low-grade inflammation within the liver tissue, leading to hepatocytes’ lipid overload and fibrosis [10, 11]. Additionally, it may lead to an increased risk of cardiovascular events occurrence, formulating a vicious cycle between the liver and other cardiometabolic disorders [12–14]. In this context, several biomarkers of gut permeability and intestinal homeostasis have gained particular interest, including citrulline (CIT) and Intestinal Fatty Acid-Binding Protein (I-FABP). CIT is a non-essential amino acid, which may be synthesized endogenously by the human organism. This process occurs in two types of cells: hepatocytes and enterocytes. In the first mentioned cell type, it is immediately used in the urea cycle and metabolised to arginine. Therefore, it is not released into the systemic circulation and does not affect its serum level [15]. In turn, in small intestine epithelial absorptive cells, dietary amino acids (glutamine, proline, ornithine, or L-arginine) act as substrates for numerous biochemical reactions, which lead to the CIT de novo synthesis in enterocytes [16]. Later, synthesized CIT (and to a lesser extent, CIT from the dietary intake) is transported through the basolateral luminal membrane into the portal circulation, passes through the liver and reaches the systemic circulation [17]. Thus, serum citrullinemia may serve as a biomarker of small intestine enterocytes mass, with their reduced plasma levels indicating gut dysfunction or enterocytes loss [18]. I-FABP, also known as a Fatty Acid-Binding Protein 2 (FABP2), is an isoform of FABPs specifically expressed in epithelial cells of the small intestine mucosa and located on the intestinal villi. It is engaged in the dietary lipids transfer from the intestinal lumen to enterocytes. Additionally, I-FABP binds intracellular redundant unesterified fatty acids, preventing enterocytes from lipotoxicity [19]. In states of the enterocytes damage, a soluble I-FABP form is released extracellularly into the plasma [20]. Therefore, its concentration may reflect the level of intestinal wall dysfunction or intestinal permeability issue.

**Fig. 1:**
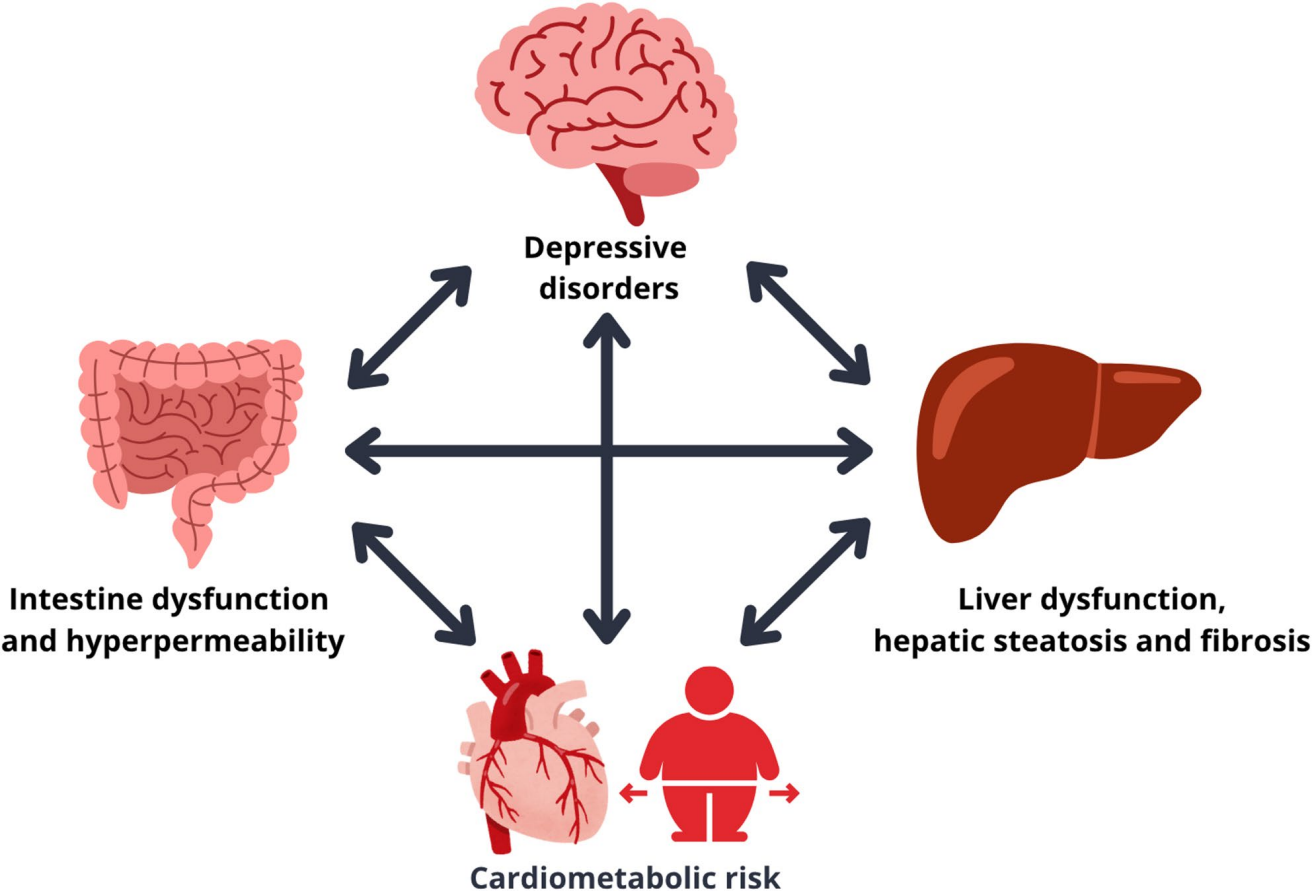
Complex and multidirectional network of dependencies between depressive disorders, intestine dysfunction, liver abnormalities and cardiometabolic risk, as a rationale for this research

Alongside these gut-related biomarkers, non-invasive hepatic indices such as the alanine transaminase to aspartate aminotransferase ratio (ALT/AST ratio), Framingham Steatosis Index (FSI), Hepatic Steatosis Index (HSI), AST to Platelet Ratio Index (APRI), and Fibrosis-4 (FIB-4) Index have become increasingly relevant in clinical and research settings. These biomarkers allow for the early detection and monitoring of hepatic abnormalities, without the need for performing invasive procedures [21, 22]. Additionally, it has been revealed that they are associated with the overall cardiovascular risk (CVR) [23, 24]. Nevertheless, hepatic alterations in individuals with depression, particularly in the context of combined metabolic and enteric dysfunction, remain insufficiently investigated. (Fig. 1). Therefore, studies integrating non-invasive liver indices with biomarkers of gut permeability and intestinal homeostasis may help elucidate mechanisms linking DD, metabolic alterations, and hepatic dysfunction, while also providing new insights into potential interventional strategies.

### The aim of the study

To make up for the gap of knowledge in the field, we aim to investigate the associations between the non-invasive hepatic steatosis and fibrosis indices and biomarkers of intestinal dysfunction in patients diagnosed with DD, taking into account the presence of MetS or its components, as well as dietary, behavioral and psychosocial factors, and antidepressant intake status.

### Hypothesis of the study

We hypothesize that the abnormal function of the liver (measured by FSI, HSI, APRI, FIB-4), as well as cardiovascular risk (reflected in APRI and FIB-4), may be associated with altered serum levels of intestinal dysfunction biomarkers, such as CIT and I-FABP, in patients with DD. Metabolic, dietary, and psychometric factors may further contribute to these associations.

## Material and methods

### Study design

This is a cross-sectional observational study, reported according to the Strengthening the Reporting of Observational studies in Epidemiology (STROBE) statement guidelines [25].

### Study population

A study population consists of 116 subjects primarily enrolled into the PRO-DEMET randomized controlled trial, assessing the influence of probiotic supplementation on depressive symptoms, inflammation, and oxidative stress parameters and fecal microbiota in patients with depression depending on metabolic syndrome comorbidity (ClinicalTrials.gov identifier: NCT04756544) [26]. Patients were recruited into this study between December 2020 and May 2023, in the lodzkie voivodship and surroundings (Poland), with the help of social media advertisements, posters or leaflets placed in the outpatient clinics, as well as the snowball method. The trial was conducted in accordance with the Consort guidelines.

#### Inclusion and exclusion criteria

Age above 18 years old, the diagnosis of depressive disorders, according to the 11th International Classification of Diseases [27], as well as no change in the antidepressant and antianxiety medications application three weeks prior to the beginning of the study had to be fulfilled by potential study participants, to be enrolled into this research. A minimum of 13 points in the Montgomery-Åsberg Depression Rating Scale (MADRS) was set as the inclusion criterion [26].

Waist circumference values (88 cm for women, and 102 cm for men) were applied for the abdominal obesity diagnosis [28]. To diagnose the MetS, the Polish 2022 Metabolic Syndrome Definition [28] criteria were used.

The following exclusion criteria were established: pregnancy; current exacerbated severe somatic disease; an acute phase of infection and/or treatment with antibiotics; vaccination; the diagnosis and/or the presence of symptoms of autoimmune diseases, serious immunocompromise status, inflammatory bowel diseases, cancer, IgE-dependent allergy; a significant change in dietary habits; a significant change in dietary supplementation; a significant change in daily physical activity; a significant change in cigarette smoking pattern; a significant change in the treatment regimen with proton-pump inhibitors, metformin, laxatives, systemic steroids, nonsteroidal anti-inflammatory drugs, antipsychotics, or any other medications influencing the microbiota in the previous four weeks; supplementation with probiotics or prebiotics; psychiatric comorbidities (except specific personality disorders, anxiety disorders, and caffeine or nicotine addiction) and high risk of the suicide attempt; any major neurological disorder or any medical disability that may interfere with a subject’s ability to complete the study procedures; current or recent participation in another research study involving an intervention that may alter outcomes relevant to this study.

#### Sample size

The sample size was calculated in previously performed study, where the number of enrolled subjects was the same [29].

#### The risk of bias

To minimize the risk of selection bias during the study subjects’ enrollment, recall bias when using study questionnaires, or interviewer bias, several solutions were provided. They are described in another related study [20].

### Outcome measures

Primary, secondary, and tertiary outcome measures have been presented in Table 1.

**Table 1:** Study outcome measures

Outcome measures	Description
primary	CIT, I-FABP, I-FABP/CIT ratio
secondary	ALT, AST, ALT/AST ratio, FSI, HSI, APRI, FIB-4
tertiary	antidepressant treatment, BMI, CHOL, CRP, DASS, dBP, dietary habits, dietary supplements, fGlc, HDL-c, LDL-c, MADRS, NLR, non-HDL-c, physical activity level, PLT, PLR, sBP, SII, TNF-α, TGs, WC, QoL.

*Abbreviations* *ALT* Alanine transaminase, *APRI* AST-to-platelet ratio index, *AST* Aspartate aminotransferase, *BMI* Body mass index, *CHOL* Cholesterol, *CIT* Citrulline, *CRP* C-reactive protein, *DASS* Depression, Anxiety and Stress Scale, *dBP* diastolic blood pressure, *fGlc* fasting serum glucose, *FIB-4* fibrosis-4 index, *FSI* Framingham Steatosis Index, *HDL-c* High-density lipoprotein cholesterol, *HSI* Hepatic steatosis index, *I-FABP* Intestinal fatty acid binding protein, *LDL-c* Low-density lipoprotein cholesterol, *MADRS* The Montgomery-Åsberg Depression Rating Scale, *NLR* Neutrophil to lymphocyte ratio, *non-HDL-c* non-high density lipoprotein cholesterol, *PLT* Platelet count, *PLR* Platelet to lymphocyte ratio, *sBP* systolic blood pressure, *SII* Systemic immune-inflammation index, *TGs* triglycerides, *TNF*-α Tumor necrosis factor-alpha, *WC* Waist circumference, *QoL* quality of life

### Biological material collection. Laboratory measurements

Fasting venous blood samples were collected from each subject between the 8:00 a.m. and 11:00 a.m. by qualified nurses. Basic laboratory analyses were performed in the Department of Laboratory Diagnostics, Central Teaching Hospital, Medical University of Lodz, Poland. Serum was obtained by centrifugation process of the whole blood collected in tubes containing clotting activator. After centrifugation the serum was aliquoted and stored at − 80 °C for further use.

To assess the presence of the intestine dysfunction in general, serum levels measurements of two specific biomarkers (CIT and I-FABP), were performed. Serum CIT levels were assessed in the Institute of Food Technology and Analysis, Faculty of Biotechnology and Food Sciences, Lodz University of Technology, Poland, using the Metamino^®^ kit. I-FABP was analyzed in the Department of Biomedicine and Genetics, Medical University of Lodz, Poland, with the use of the commercial ELISA test for Fatty Acid-Binding Protein, intestinal (cat no. E0559h, EIAab, Wuhan China). The exact methodology process of the CIT and I-FABP serum levels assessment is described in the Supplementary Materials.

Moreover, I-FABP/CIT ratio, as another alternative intestine dysfunction and intestinal enterocyte loss index, was also calculated [30].

### Anthropometric and clinical measurements

Physical examination, including body weight, height, blood pressure, and waist circumference measurements, was performed on each subject by the medical doctor (principal investigator, PI).

### Non-invasive hepatic steatosis and liver fibrosis biomarkers

Based on the obtained data, following non-invasive hepatic steatosis biomarkers were calculated:

- ALT/AST ratio, with the cut-off point at 1.33 [31]:

$$\:\frac{ALT}{AST}ratio=\:\frac{\left[ALT\:value\right]}{\left[AST\:value\right]}$$

- Framingham Steatosis Index (FSI), and the cut-off point for this mean is accepted as −1.2 [32]:

$$\begin{aligned}{c}\:FSI\:&=\:-7.981+0.011\bullet\:age\:\left[in\:years\right]-0.146\bullet\:sex\:\left[0=male,\:1=female\right]+0.173\bullet\:\:\:BMI\:\left[\frac{kg}{m2}\right]+0.007\bullet\:triglicycerides\:\left[\frac{mg}{dl}\right]+0.593\bullet\:hypertension\:\left[0=no,\:1=yes\right]+0.789\bullet\:diabetes\:mellitus\:\left[0=no,\:1=yes\right]+1.11\bullet\:\frac{ALT}{AST}\:ratio\:\geq\:1.33\:\lbrack0=no,\:1=yes\rbrack\end{aligned}$$

- Hepatic Steatosis Index (HSI), with the cut-off point at 36, defined as a following calculations score [33, 34]:

$$\begin{aligned}\:HSI&=8\bullet\:\frac{ALT}{AST}\:ratio+BMI\:\left[\frac{kg}{m2}\right]+2\:\left(if\:type\:2\:diabetes\:mellitus\right)+2\:\left(if\:female\right)\end{aligned}$$

Additionally, several liver fibrosis biomarkers were used in these analyses, which also have been found to be indirect but reliable indicators of the cardiovascular risk (CVR):

- AST to Platelet Ratio Index (APRI), with the estimated cut-off point at 0.5 [35, 36]:

$$\:APRI=\frac{\frac{\left[AST\:value\right]}{\left[AST\:upper\:limit\:of\:normal\right]}}{\left[Platelet\:count\right]}\:\bullet\:100$$

- Fibrosis-4 Index (FIB-4) – another hepatic fibrosis index, with the cut-off point at 1.3 [37]:

$$\:FIB-4=\frac{age\:\left[in\:years\right]\:\bullet\:\left[AST\:value\right]}{\left[Platelet\:count\right]\bullet\:\sqrt{\left[ALT\:value\right]}}\:$$

### Questionnaires and scales

Initially, the demographic, lifestyle, and health-related data questionnaire was performed. The exclusion criteria were examined. In order to obtain data regarding dietary habits, Food Frequency Questionnaire (FFQ-6) by Wądłowska was used – dietary intake index was calculated as an average consumption of all products within a given category as assessed on a scale of 1–6: 1 – never or almost never; 2 – once a month; 3 – several times a month; 4 – several times a week; 5 – every day; 6 – several times a day [38]. Additionally, to assess the level of depressiveness and anxiety, the Montgomery-Åsberg Depression Rating Scale (MADRS) [39] and the Depression, Anxiety and Stress Scale (DASS) [40] were applied. The World Health Organization Quality-of-Life Scale (WHOQOL-BREF) was used to assess study subjects’ quality of life [41]. Furthermore, the level of physical activity was measured with the International Physical Activity Questionnaire (IPAQ) and expressed as metabolic equivalent (MET)-minutes per week [42].

### Ethics

All subjects gave their informed consent to participate in this study. The research was conducted in accordance with the Declaration of Helsinki. Additionally, the protocol of the parental PRO-DEMET randomized controlled trial was approved by the Bioethical Commission of the Medical University of Lodz, Poland (15th December 2020; reference number RNN/228/20/KE).

### Statistical analyses

The distribution of continuous variables was assessed using the Shapiro–Wilk test. Differences among normally distributed data were analyzed using Welch’s t-test for comparisons between two groups or ANOVA for more than two groups, with results reported as means with standard deviations (SD). For non-normally distributed data, the Mann–Whitney U test was used for two groups, with effect sizes reported as rank-biserial correlations. The Kruskal–Wallis test was applied for more than two groups, with results presented as medians with interquartile ranges (IQR). In post-hoc analyses, Tukey’s HSD test was employed for variables significant in ANOVA, whereas the Conover–Iman test with Holm-Bonferroni correction was utilized for variables significant in the Kruskal–Wallis test. Spearman’s rank correlation coefficient (ρ) was used for correlation analysis due to the non-normal distribution of the analyzed variables. Based on the concept of immunometabolic depresssion, as well as above-mentioned hypotheses of this study, logistic regression with forward selection procedure was applied to detect predictors of increased FIB-4, which is indicative of increased risk of liver fibrosis, while multiple linear regression (MLR) with forward selection procedure was used to create a model explaining variance of HSI, that is used as a screening tool for liver steatosis. To evaluate multicollinearity prior to model building, variance inflation factors (VIFs) were calculated for all independent variables. A VIF threshold of 5 was used to identify potentially collinear predictors. All predictors in our study showed low collinearity (maximum VIF = 1.56). To assess the explanatory power of the regression model, Nagelkerke’s R² was calculated.

All statistical analyses were conducted using Python version 3.11.5 with the SciPy package (version 1.14.1) as well as Statistica 13.1 (TIBCO Software Inc., USA), and p-values less than 0.05 were considered statistically significant (Bonferroni correction was applied to adjust for multiple comparisons)

## Results

### Study population characteristics

Study population characteristics are presented in the Table 2.

**Table 2:** Study population characteristics. Data is shown as ratio, number of cases with percentages (n (%)), mean with standard deviation (M ± SD) or median with interquartile range (Mdn (IQR))

Sample characteristics analyzed parameter		Ratio / Number (%) / Mean ± SD / Median (IQR)	Missing data (%)
Basic parameters			
Sex (F: M)		99:17	0
Age (years)		32.6 (22.6–42.3)	0
Diagnosis according to ICD-11 (6A70/6A71:6A72/6A73)		42:74	0
Psychotropic medications		81 (69.8)	0
Antidepressants		81 (69.8)	0
Antipsychotics		6 (5.2)	0
Comorbidities		61 (52.6)	0
Different than psychotropic pharmacological treatment		41 (35.3)	0
Smoking cigarettes		18 (15.5)	0
Dietary supplements intake		59 (50.9)	0
Physical activity (MET-min/week)		1804.0 (1084–3012)	64.66
Liver-related non-invasive biomarkers			
AST (U/l)		22.5 (19.1–25.9)	0
ALT (U/l)		17.0 (13.0–24.3)	0
ALT > 25.0 (F) or ALT > 33.0 (M)		22.0 (19.0)	NA
ALT/AST		0.8 (0.6–0.9)	0
ALT/AST ≥ 1.33		10.0 (8.6)	NA
HSI		32.7 (28.7–36.4)	0
HSI > 36.0		31 (26.7)	NA
FSI		−2.7 (−3.5–−1.6)	0
FSI > −1.2		18.0 (15.5)	NA
APRI		0.2 (0.2–0.3)	0
APRI > 0.5		3.0 (2.6)	NA
FIB-4		0.6 (0.5–0.8)	0
FIB-4 > 1.3		9.0 (7.8)	NA
Dietary habits index			
Sweets and snacks		2.6 ± 0.7	4.31
Diary and eggs		3.2 (2.7–3.6)	4.31
Cereal products		3.2 (2.8–3.4)	4.31
Oils		2.6 ± 0.6	5.17
Fruits		2.8 ± 0.5	4.31
Vegetables and seeds		3.4 ± 0.6	4.31
Meat (including fish)		2.2 (1.9–2.8)	4.31
Drinks (excluding water)		2.0 (1.7–2.4)	4.31
Alcoholic beverages		1.7 (1.3–2.3)	4.31
Processed food products		2.3 (2.1–2.7)	4.31
Psychometric parameters			
MADRS total score		19.5 (17.0–24.0)	0
DASS total score		64.1 ± 22.1	4.31
DASS subdomains	Depression	21.0 (15.0–28.0)	4.31
	Anxiety	17.0 (10.0–23.0)	4.31
	Stress	24.0 (20.0–34.0)	4.31
QoL total score		73.2 ± 12.3	4.31
QoL subdomains	Physical	18.8 ± 4	4.31
	Psychological	15.3 ± 3.6	4.31
	Social	8.4 ± 2.4	4.31
	Environment	25.3 ± 4.9	4.31
Metabolic parameters			
MetS diagnosis according to Polish guidelines		30.0 (25.9)	NA
Weight (kg)		68.8 (59.8–81.2)	0
BMI (kg/m^2^)		24.5 (21.3–27.5)	0
WC (cm)		85.0 (74.0–95.2)	0
sBP (mmHg)		121.0 (111.0–129.2)	0
dBP (mmHg)		82.3 (8.6)	0
fGlc (mmol/l)		5.1 (4.9–5.4)	0
cholesterol (mmol/l)		5.4 (1.1)	0
LDL-c (mmol/l)		3.3 (0.9)	0
HDL-c (mmol/l)		1.6 (0.4)	0
non-HDL-c (mmol/l)		3.8 (1.0)	0
TG (mmol/l)		1.0 (0.8–1.4)	0
Inflammation parameters			
CRP (mg/l)		1.2 (0.5–2.9)	0
TNF-α (pg/ml)		5.8 (2.0–7.2)	0.86
NLR		1.3 (0.9–1.8)	0.86
PLR		143.1 (117.3–168.4)	1.72
SII		351.9 (251.2–491.5)	0.86
Microbiota-gut parameters			
CIT (µmol/L)		45.9 (33.8–55.1)	0.86
I-FABP (pg/ml)		1821.3 (1184.9–2392.4)	1.72
I-FABP/CIT ratio		41.3 (23.1–63.0)	1.72

*Abbreviations:* *6A70* depressive episode, *6A71* recurrent depression, *6A72* dysthymia, *6A73* mixed depressive and anxiety disorder, *ALT* Alanine transaminase, *APRI* AST-to-platelet ratio index, *AST* Aspartate aminotransferase, *BMI* Body mass index, *CIT* citrulline, *CRP* C-reactive protein, *DASS* Depression, Anxiety and Stress Scale, *dBP* diastolic blood pressure, *F* women, *fGlc* fasting serum glucose, *FIB-4* Fibrosis-4 index, *FSI* Framingham Steatosis Index, *HDL-c* High-density lipoprotein cholesterol, *HSI* Hepatic steatosis index, *I-FABP* Intestinal fatty acid binding protein, *IQR* Interquartile range, *M* Men, *MADRS* The Montgomery-Åsberg Depression Rating Scale, *Mdn* median, *M* ± *SD* mean with standard deviation, *NA* Not applicable, *NLR* Neutrophil to lymphocyte ratio, *non-HDL-c* non-high density lipoprotein cholesterol, *PLR* Platelet to lymphocyte ratio, *sBP* systolic blood pressure, *SII* Systemic immune-inflammation index, *TGs* Triglycerides, *TNF*-α Tumor necrosis factor-alpha, *WC* Waist circumference, *QoL* Quality of life

After the critical evaluation of the obtained data, one participant was excluded from the further analyses as her I-FABP result value (10127 pg/ml) was an outlier, as well as above the established cut-off points, suggesting probable serious form of enteropathy or acute intestinal inflammatory state occurrence [43]

### The associations between serum levels of CIT, I-FABP and I-FABP/CIT ratio, and non-invasive liver-related parameters in study population

**Fig. 2:**
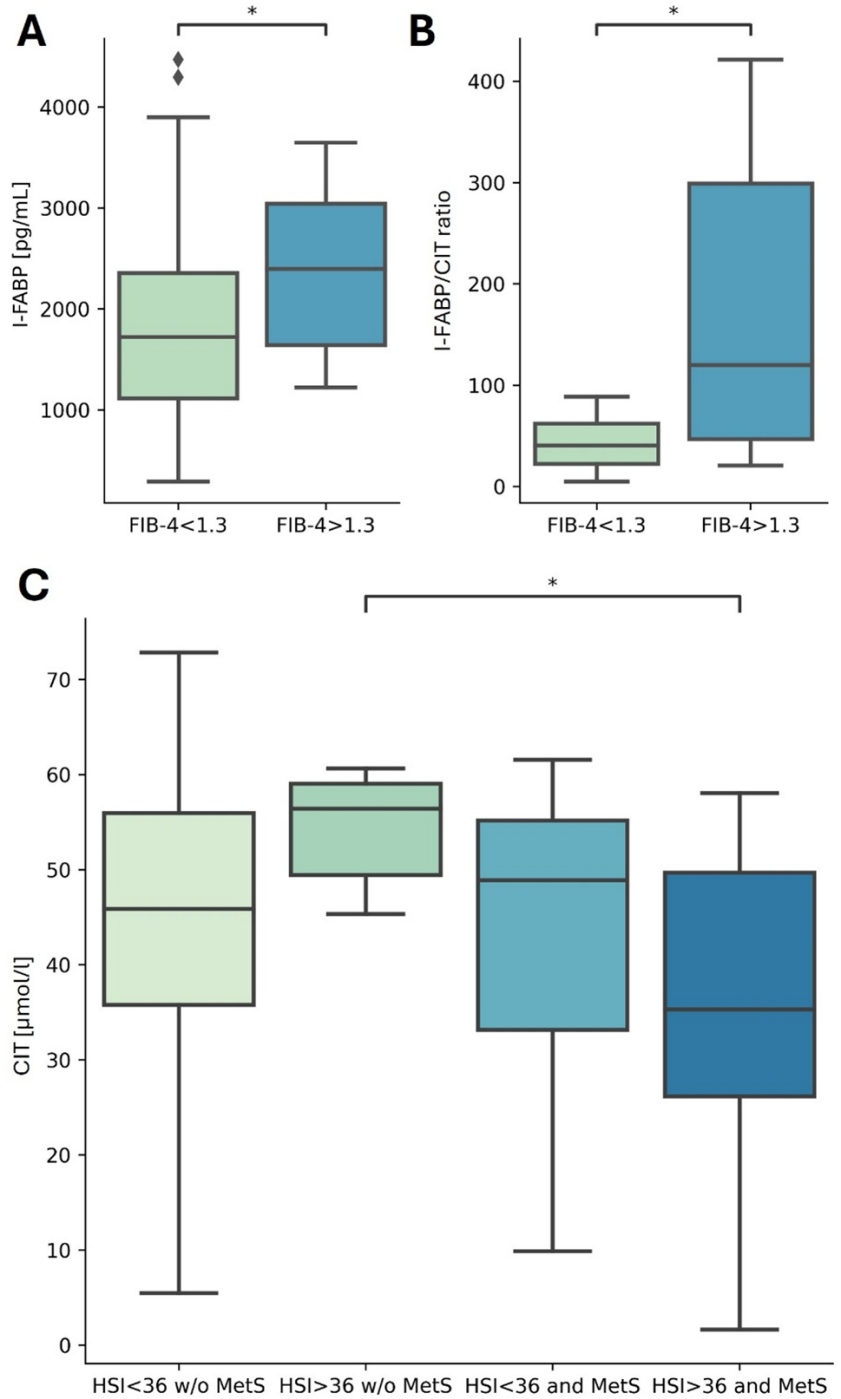
Box and whisker plots for the following analyses: **A** serum I-FABP levels, depending on the FIB-4 > 1.3 (yes/no), **B** I-FABP/CIT ratio, depending on the FIB-4 > 1.3 (yes/no), **C** association between serum CIT levels and HSI > 36.0 (yes/no) values, depending on the MetS presence. * - *p* < 0.05

First, the analysis of associations between serum levels of CIT, I-FABP, and non-invasive hepatic steatosis and fibrosis indices, regarding their cut-off points indicating specific liver abnormalities, was performed. It revealed higher median values of I-FABP and I-FABP/CIT ratio in subjects with FIB-4 values above the 1.3 cut-off point, than in those with FIB-4 < 1.3 (Fig. 2 A and B). There were no differences between subgroups, when stratified by other liver-related indices (Table 3.)

**Table 3:** Serum levels of CIT and I-FABP, between subgroups with within normal range vs. above normal range parameters: ALT > 25 (women) or ALT > 33 (men) (yes/no), hepatic steatosis – HSI > 36 (yes/no), ALT/AST > 1.33 (yes/no), FSI > −1.2 (yes/no), and possible liver fibrosis – FIB-4 > 1.3 (yes/no), APRI > 0.5 (yes/no). Data is presented in median with interquartile range (IQR). In **bold –** *p* < 0.05

	ALT above the reference range (*N* = 22)	ALT within the reference range (*N* = 93)	*p*	p*	*r*_rb_
CIT (µmol/l)	44.0 (29.8–53.2)	46.3 (34.9–55.6)	0.502	0.999	− 0.093
I-FABP (pg/ml)	1837.8 (1441.6–2384.6)	1725.5 (1110.3–2374.8)	0.471	0.999	0.100
I-FABP/CIT ratio	43.7 (32.1–62.8)	40.1 (20.7–62.9)	0.239	0.717	0.163
	ALT/AST above the reference range (*N* = 10)	ALT/AST within the reference range (*N* = 105)	p	p*	*r*_rb_
CIT (µmol/l)	50.2 (37.4–57.2)	45.6 (33.6–54.6)	0.578	0.999	0.108
I-FABP (pg/ml)	1560.4 (1240.3–2222.0)	1832.1 (1183.1–2389.0)	0.762	0.999	− 0.059
I-FABP/CIT ratio	40.1 (20.7–51.7)	41.3 (23.1–63.0)	0.883	0.999	− 0.029
	HSI above the reference range (*N* = 31)	HSI within the reference range (*N* = 84)	p	p*	*r*_rb_
CIT (µmol/l)	45.3 (29.7–53.6)	46.2 (35.6–55.7)	0.330	0.990	− 0.119
I-FABP (pg/ml)	2214.6 (1553.9–2412.5)	1636.8 (1107.6–2214.6)	0.097	0.291	0.203
I-FABP/CIT ratio	45.0 (36.5–76.8)	37.6 (21.0–60.8)	0.085	0.255	0.211
	FSI above the reference range (*N* = 18)	FSI within the reference range (*N* = 97)	p	p*	*r*_rb_
CIT (µmol/l)	39.5 (29.8–51.1)	46.3 (34.9–56.0)	0.168	0.504	− 0.206
I-FABP (pg/ml)	1972.2 (1441.6–2392.4)	1725.5 (1142.4–2365.4)	0.541	0.999	0.092
I-FABP/CIT ratio	45.8 (38.8–66.2)	38.8 (22.1–62.6)	0.189	0.567	0.196
	APRI above the reference range (*N* = 3)	APRI within the reference range (*N* = 112)	p	p*	*r*_rb_
CIT (µmol/l)	40.0 (37.8–45.4)	46.2 (33.5–55.7)	0.658	0.999	− 0.153
I-FABP (pg/ml)	1511.2 (1214.8–1998.3)	1821.3 (1184.9–2378.6)	0.782	0.999	− 0.097
I-FABP/CIT ratio	42.4 (30.3–52.3)	41.2 (23.1–63.0)	0.940	0.999	− 0.03
	FIB-4 above the reference range (*N* = 9)	FIB-4 within the reference range (*N* = 106)	p	p*	*r*_rb_
CIT (µmol/l)	30.4 (7.8–51.3)	46.5 (35.1–56.0)	0.085	0.255	− 0.348
I-FABP (pg/ml)	**2395.8 (1642.6**–**3042.9)**	**1723.3 (1112.9**–**2354.1)**	**0.027**	0.081	0.447
I-FABP/CIT ratio	**120.0 (46.7**–**299.1)**	**40.4 (22.3**–**62.2)**	**0.018**	0.054	0.479

*Abbreviations*: *ALT* Alanine transaminase, *APRI* AST-to-platelet ratio index, *AST* Aspartate aminotransferase, *CIT* Citrulline, *FIB-4* Fibrosis-4 index, *FSI* Framingham Steatosis Index, *HSI* Hepatic steatosis index, *I-FABP* Intestinal fatty acid binding protein, *r*_rb_ Rank-biserial correlation, p*—the p-value after Bonferroni correction

### Correlation analyses of the CIT, I-FABP and I-FABP/CIT ratio values with non-invasive liver steatosis and fibrosis markers

The correlation analysis of the citrulline and I-FABP serum levels, as well as I-FABP/CIT ratio values, with non-invasive liver steatosis and fibrosis biomarkers did not reveal any significant relationship besides the weak positive correlation between the APRI score and serum I-FABP level (ρ = 0.15, *p* = 0.021) (Table S1)

### The subgroup analyses of CIT, I-FABP, and I-FABP/CIT ratio values, depending on basic and metabolic parameters

Next, analyses in subgroups, depending on the (1) sex, (2) presence of the abdominal obesity, (3) MetS diagnosis, (4) antidepressants intake, (5) diagnosis of the specific mental disorder (depressive episode vs. mixed depressive and anxiety disorder), (6) supplements intake, (7) smoking status and (8) comorbidities were conducted (Table S2).

In Post hoc analysis, subjects with HSI > 36.0 and without a diagnosis of concomitant MetS had higher CIT levels compared to those meeting both of the above-mentioned criteria (Mdn (IQR): 56.4 (49.4–59.0) vs. 35.285 (26.2–49.7), respectively, p_adj_=0.016, r_rb_=0.529) (Fig. 2 C).

### The whole model approach

To build a model of a relationship between liver-related biomarkers and intestine dysfunction indicators, a multiple logistic regression was conducted, with CIT, WC, QoL, vegetables and seeds consumption, male sex, and psychiatric diagnosis as the predictors, with FIB-4 > 1.3 value as the dependent variable. The model was statistically significant, X^2^(6) = 21.77, *p* = 0.001. The model demonstrated a satisfactory goodness of fit and explained 41.5% of the variance (Nagelkerke R² = 0.415). Multicollinearity was not detected (VIF < 1.5 for all predictors). Vegetable and seed consumption, CIT levels, WC and QoL reached statistical significance in the model. WC demonstrated a consistent association (*p* =.019, OR = 1.1; 95% CI: 1.017-1.017.017,205), with a small effect size (10% increase in odds of FIB-4 being over 1.3 per 1 cm increase in WC). Vegetable&seeds domain in dietary pattern was a protective predictor (*p* = 0.030, OR = 0.152; 95% CI: 0.026–0.875) with moderate effect size (85% decrease in odds of FIB-4 > 1.3 per 1 point increase in Vegetable&seeds subscale). As for other predictors, the direction of their effect remained uncertain. However, for CIT, each 1 µmol/L increase was associated with a 4.1% decrease in the risk of elevated FIB-4 (OR = 0.959; 95% CI: 0.921–0.999).

Regarding HSI, linear regression models did not identify CIT or I-FABP as significant predictors. However, it was shown that WC (B = 0.687, t = 10.483, *p* < 0.001), fGlc (B = 0.141, t = 2.373, *p* = 0.019) and TG (B = 0.155, t = 2.575, *p* = 0.011) levels were significant positive predictors of HSI (F(4,110) = 63.587, *p* < 0.001, Adjusted R^2^ = 0.687).

## Discussion 

Liver dysfunction is not a rare phenomenon among individuals diagnosed with DD [44, 45]. It is postulated that at the core of this state may lie an immunometabolic systemic dysfunction, with the interplaying role of intestine dysfunction and gut hyperpermeability [46]. Indeed, the study results shed light on the intriguing and complex network of dependencies between non-invasive liver-related indices and serum concentrations of intestinal function biomarkers. 

### Associations of intestinal dysfunction indicators with non-invasive liver-related biomarkers 

First, we observed higher levels of I-FABP and an increased I-FABP/CIT ratio in individuals with depression whose calculated FIB-4 values exceeded the cut-off point, compared to those with FIB-4 < 1.3. Although the corrected p values did not reach statistical significance, the rank-biserial correlations indicated moderate effect sizes, thus, suggesting a potentially meaningful difference in intestinal barrier biomarker levels between the subgroups. 

Besides the ability of FIB-4 to predict liver dysfunction and hepatic fibrosis in patients with confirmed liver disease, this biomarker has also been found to be a reliable indicator in the cardiovascular risk (CVR) assessment [47, 48]. Therefore, these findings may be interpreted within a broader scope. Numerous studies have already explored the link between liver dysfunction and intestinal mucosal injury with suspected microbial translocation into the portal circulation, highlighting their significant clinical connection, and thus, confirming our study results [49, 50]. Abdelhaleem et al. reported a positive correlation between I-FABP and FIB-4 values in patients co-infected with hepatitis C virus and human immunodeficiency virus, suggesting greater severity of liver disease with increasing I-FABP [51]. In turn, Miuma et al. showed that serum I-FABP levels tended to rise as hepatic reserve declined, as measured by the Child–Turcotte–Pugh grade and the Model for End-Stage Liver Disease score, in patients with liver cirrhosis [52]. On the other hand, both FIB-4 and I-FABP emerged as biomarkers of the CVR assessment, especially among those subjects diagnosed with metabolic or liver disorders [53–55]. Therefore, their combination may reveal a subpopulation of individuals with DD, particularly vulnerable to the long-term cardiovascular complications – future prospective studies are needed to verify this thesis. 

We have not found any statistically significant differences in CIT, I-FABP or I-FABP/CIT ratio values, in terms of other non-invasive liver-related indices, in the whole study group. We hypothesize that the heterogeneity of the study group limited the ability to observe any significant differences regarding established cut-off points for each liver abnormality indicator. Additionally, we suspect that liver biomarkers may be associated with the intestinal dysfunction only indirectly, rather than through a direct causal relationship (discussed in the next subsection). Nevertheless, we observed that median I-FABP levels were non-significantly, and with a small effect size, higher among the subgroup of study subjects with HSI > 36.0, in comparison to those with HSI < 36.0. Similar results were found regarding I-FABP/CIT ratio values. We believe that such results should be considered as a rationale for future studies, especially with larger sample sizes or more homogeneous populations, as these findings are in accordance with other research conclusions, suggesting that there is a link between the intestinal injury and hepatic steatogenesis, or liver dysfunction in general [56, 57]. 

### The subgroup analyses of intestinal dysfunction parameters, regarding basic and metabolic variables

Subgroup analysis revealed that individuals with HSI > 36.0 and no diagnosis of concomitant MetS had higher CIT levels compared to those who met both criteria. Previous studies have suggested that elevated plasma CIT may stem from impaired hepatic amino acid metabolism associated with diet-induced liver dysfunction, independent of CIT intestinal synthesis or conversion from arginine, as well as the presence of other MetS components like insulin resistance or diabetes mellitus. Interestingly, it was also revealed that elevated CIT levels may act as a predictor of the MetS development [58]. Similar findings, highlighting impaired CIT turnover accompanied by its elevated plasma levels, have also been described regarding other liver pathologies, including alcoholic steatohepatitis, cirrhosis, as well as acute liver failure [59, 60]. Conversely, Lalande et al. reported that reduced plasma CIT levels, indicating decreased functional enterocyte mass, were most pronounced in individuals with advanced glucose homeostasis alterations, independent of glomerular filtration rate, comparing to healthy controls subjects [61]. Similar findings were also reported by Gawlik-Kotelnicka et al. in individuals diagnosed with depression and concomitant MetS [62].

Although fatty liver disease and MetS often co-exist, there is existing evidence confirming that hepatic steatosis may precede the formal diagnosis of MetS and its full-blown form among some individuals [63–66]. Therefore, our observations highlight complex interactions between the gut, liver, and metabolic dysregulation. Hypothetically, the presence of MetS or their particular components, like insulin resistance, may impair gut cellular integrity and overall enterocyte mass more severely than liver dysfunction alone, leading to overall decrease in circulating CIT levels, even despite dysregulated hepatic amino acid metabolism. In contrast, subjects with hepatic impairment, in the absence of metabolic alterations, may have preserved gut integrity and homeostasis, as indicated by higher CIT concentrations, an effect potentially amplified by impaired amino acid turnover. Overall, considering the cross-sectional design of this research, such findings should be interpreted with caution, and next longitudinal studies are strongly needed to clarify the direction of such associations. 

### Determinants of association between serum CIT, I-FABP levels, and liver abnormalities indices

To identify potential determinants of liver-related biomarker elevation, a multiple logistic regression was conducted, with the FIB-4 > 1.3 as the outcome variable. Among significant predictors, higher vegetable and seed consumption, as well as higher CIT levels, were significantly associated with reduced odds of elevated FIB-4 score. Conversely, higher QoL and WC were significantly linked with increased odds of elevated FIB-4 Index values. Nevertheless, only WC and the vegetables & seeds domain index of the dietary pattern achieved odds ratios of sufficient magnitude to suggest clear clinical relevance. For CIT, a 1 µmol/L increase in CIT might be associated with a slight decrease in the risk of elevated FIB-4, although this finding should be interpreted with caution. 

All in all, such results align with the previous research suggesting that plant-based diet, as well-preserving gut cellular integrity, may minimize the risk of liver dysfunction through complex mechanisms related to the gut-liver axis, e.g. reduced intestinal permeability [67–69]. In turn, the observed association with WC is consistent with current scientific knowledge indicating that WC is a reliable marker of central obesity and increased overall cardiometabolic risk, and its contribution to the liver dysfunction, including the increased of hepatic steatosis and fibrosis, seems to be undeniable [70–72]. The positive association between QoL and FIB-4 seems to be counterintuitive and may reflect confounding or reverse causality, which need further investigation in next studies. 

Although CIT was incorporated into the linear regression model, conventional metabolic parameters appeared to have greater explanatory power for HSI, with WC exerting the strongest influence on HSI variance. Overall, it seems that the risk of fatty liver disease is more dependent on metabolic alterations indicators, rather than gut-derived signals. Nevertheless, there is lack of existing evidence which may prove this hypothesis. Moreover, it is difficult to divide gut dysfunction from metabolic disorders, as they often co-occur and influence each other, thus, the interpretation of such results becomes even more difficult. 

### Strengths and limitations of the study

The main strength of this research lies in its novelty – to the best of our knowledge, this is the first study exploring associations between I-FABP, CIT, and non-invasive liver-related parameters, especially in the subgroup of patients diagnosed with DD. The findings offer new insights into the complex interplay between gut cellular integrity, liver dysfunction, metabolic alterations and mental disorders, serving as a rationale for future empirical research in this area. 

According to the protocol of the parental PRO-DEMET research, the present study included only patients with depression, both with and without Metabolic Syndrome. Therefore, a healthy control group was not part of the sample. Moreover, sample size, especially in subgroup analyses, was relatively modest, which may have affected the statistical power of some analyses. Additionally, as the study group was derived from the PRO-DEMET cohort, the selection bias (e.c. self-selection bias) may have occurred. The timing of I-FABP and CIT measurements is not guaranteed to be representative. Although none of the participants met the criteria of advanced kidney disease based on the research exclusion criteria, a physiologic decline in renal function may have promoted higher plasma CIT levels. Moreover, precursors of CIT were not measured. Furthermore, non-invasive hepatic steatosis and fibrosis indices were used as general markers of hepatic dysfunction and indicators of cardiovascular risk. Imaging modalities such as ultrasound or FibroScan were not performed, thus, a definitive diagnosis of fatty liver disease or hepatic fibrosis could not be established. Another limitation of the present study is the absence of additional biomarkers that could provide deeper mechanistic insights into gut permeability and metabolic homeostasis in individuals diagnosed with DD. The inclusion of indicators such as lipopolysaccharide, lipopolysaccharide-binding protein, zonulin, soluble CD14 glycoprotein and short-chain fatty acids, among other metabolism-related parameters, would have strengthened the mechanistic interpretation of the observed associations. Incorporating these parameters in future studies would enable a more comprehensive analysis of the underlying pathophysiological pathways, thereby advancing the findings from simple correlations toward causal inference. Lastly, this study is cross-sectional in nature and represents a secondary analysis of a primary PRO-DEMT trial; therefore, the results of this research cannot determine cause-and-effect relationships. 

Consequently, future longitudinal studies are strongly recommended, particularly those incorporating repeated measures. Moreover, the application of time-series or mixed-effects models could help elucidate direct relationships between variables. 

## Conclusions 

The findings from this research support the hypothesis of a significant association between biomarkers of intestinal dysfunction and non-invasive liver-related parameters in individuals with DD, within the framework of the gut–liver–brain axis. The observed relationship between FIB-4 and I-FABP seems to reflect increased risk of hepatic abnormalities as well as CVR within a subpopulation of individuals with depression and suspected gut permeability alterations, with CIT as a significant predictor. On the other hand, the presence of MetS or its individual components appears to compromise gut cellular integrity and reduce enterocyte mass more profoundly than in liver dysfunction alone, as evidenced by lower circulating CIT levels. Conversely, individuals with liver dysfunction, in the absence of MetS, may have preserved intestinal function, as indicated by higher CIT concentrations. Nevertheless, further empirical studies are needed to confirm this hypothesis.

## Supplementary Information

Supplementary Material 1

Supplementary Material 2

## Data Availability

The dataset is available from the corresponding author (O.G.K.) upon the reasonable request.
