# Supplementary material for: I-FABP, citrulline and non-invasive liver dysfunction indices in patients with depression – cross-sectional study results
Source: BMC Gastroenterol. 2026 Jan 3;26:88. doi: 10.1186/s12876-025-04559-7 (PMC12871022; doi:10.1186/s12876-025-04559-7)
Supplement: Supplementary file 1 — Supplementary Material 1 [file 12876_2025_4559_MOESM1_ESM.docx]

1. Serum CIT levels assessment.

The samples were analyzed with Metamino® kit (Chromservis s.r.o., Praha, Czech Republic). 100 μL of extracted plasma was pipetted and 100 μL of precipitation medium was added and the sample was centrifuged 60 s at 1,500 ×g (6,000 rpm). 25 μL of precipitated sample was pipetted and 10 μL of solution with internal standard was added to each sample preparation vial. 25 μL of catalytic solution was pipetted into each sample preparation vial and vortexed 10 sec. Pipette 10 μL of reagent solution into each sample preparation vial and vortex 5-10 s. After 2-3 min derivatization the sorbent was activated and equilibrated in microspin filter by adding: 200 μL of MSPE activation medium, then centrifuged 60 s at 1,500 ×g (6,000 rpm). 200 μL of MSPE sorbent equilibration medium was then centrifuged 60 sec at 1,500 ×g (6,000 rpm). Derivatization reaction mixture was diluted with 400 μL of diluting and washing medium and vortexed 10s. The diluted reaction mixture (typically 500 μL) was loaded to the wetted microspin filter sorbent and left to stand for 2 min and centrifuged 60 s at 1,500 ×g (6,000 rpm). The sorbent was washed in the microspin filter with 200 μL of diluting and washing medium and centrifuge 60 s at 1,500 ×g (6,000 rpm). The microspin filter was placed into a new centrifugal vial, 200 μL of eluting medium was added and centrifuged 60 s at 1,500 ×g (6,000 rpm). The eluate was transfered into the autosampler vial. The LC=MS analysis was performed by means of the UHPLC-ESI-MS liquid chromatography method using a UHPLC chromatograph with an ESI-MS detector (Shimadzu, Kyoto, Japan). 5 μL samples were injected Metamino® LC-MS column 100 x 2.1 mm (flowrate 0.3 mL/min). The mobile phase A was 5 mmol/L ammonium formate in water, and eluent B 5 mol/L ammonium formate in methanol. The gradient used was 0–10.00 min, 55–90% B; 10.00–10.50 min, 90–100%; 10.50–12.00 min, 100–100% B; 12.00–12.01 min, 100–55% B; 12.01–15.00 min, 55.00-55.00%. ESI source was in positive ion mode. The column temperature was 35 °C, drying gas temperature was 300 °C, and capillary temperature was 500 °C. Nitrogen was used as nebulizer gas. Full mass spectra were obtained in the mass range from m/z 100 to 750 in positive ion mode. The obtained MS spectrum signal data was collected and processed with LabSolutions software.

2. Serum I-FABP levels assessment.

The serum level of the I-FABP protein was measured using a commercial ELISA kit for intestinal Fatty acid-binding protein (cat no. E0559h, EIAab, Wuhan, China), with a sensitivity detection range of 62.5-4000 pg/mL. According to the kit protocol, serum samples were diluted 4x or 8x with Sample Diluent before the assay, and aliquots of approximately 25-50 µL per patient were used in each ELISA (samples were measured in duplicate). The intensity of the colorimetric reactions, which was proportional to protein/substrate binding, was measured at 450 nm using an Elx800 plate reader (BioTek, Winooski, VT, USA). The final I-FABP concentration in serum was determined as the mean of the two measurements and calculated based on the dilution applied.

**Table S1.** Correlation coefficients for associations between CIT, I-FABP, I-FABP/CIT values and non-invasive liver steatosis and fibrosis markers. In **bold** – p < 0.05.

|  | CIT | I-FABP | I-FABP/CIT ratio |
| --- | --- | --- | --- |
| ALT | 0.0072 | 0.0708 | 0.0532 |
| ALT/AST ratio | 0.0146 | 0.0254 | 0.0243 |
| HSI | -0.0606 | 0.0052 | 0.049 |
| FSI | -0.0587 | 0.0014 | 0.0452 |
| APRI | -0.0343 | **0.1476** | 0.1 |
| FIB-4 | -0.0621 | 0.1089 | 0.0857 |

**Table S2.** Chosen (when the analysis was powered enough) interactions between intestine dysfunction indicators and liver abnormalities indices, regarding A) sex, B) presence of the abdominal obesity, C) MetS diagnosis, D) antidepressants intake, E) diagnosis of the specific mental disorder (depressive episode vs. mixed depressive and anxiety disorder), F) supplements intake, G) smoking status and H) comorbidities. Data is shown as the median with interquartile range (IQR). In **bold** – p < 0.05.

**A) sex**

| Analyzed parameter | ALT < ULN and F sex  (N = 84) | ALT > ULN  and F sex  (N = 14) | ALT < ULN and M sex  (N = 9) | ALT > ULN  and M sex  (N = 8) | p | p* |
| --- | --- | --- | --- | --- | --- | --- |
| CIT [mcmol/l] | 46.5  (35.3–55.6) | 51.7  (29.8–58.1) | 43.0  (28.2–53.7) | 37.8  (30.4–44.3) | .330 | .999 |
| I-FABP [pg/ml] | 1723.3  (1112.9–2363.1) | 2028.6 (1601.6–2731.5) | 2382.3 (886.5–2448.5) | 1560.4 (1293.5–2256.3) | .666 | .999 |
| I-FABP/CIT ratio | 39.5  (21.1–62.8) | 41.6  (27.1–60.7) | 41.6  (20.7–92.3) | 46.7  (41.3–63.4) | .657 | .999 |
| Analyzed parameter | HSI < 36.0  and F sex  (N = 76) | HSI > 36.0  and F sex  (N = 22) | HSI < 36.0  and M sex  (N = 8) | HSI > 36.0  and M sex  (N = 9) | p | p* |
| CIT [mcmol/l] | 46.5  (35.7–56.0) | 49.0  (30.5–55.7) | 42.5  (35.9–47.9) | 35.6  (17.4–49.5) | .355 | .999 |
| I-FABP [pg/ml] | 1636.8 (1112.9–2308.1) | 2233.4 (1670.4–2707.8) | 1846.7 (911.5–2481.1) | 1609.6 (1418.5–2351.0) | .242 | .999 |
| I-FABP/CIT ratio | 37.2  (20.6–62.0) | 45.8  (36.0–69.1) | 44.6  (32.0–48.2) | 44.9  (37.8–137.1) | .385 | .999 |
| Analyzed parameter | FIB-4 < 1.3 and F sex  (N = 89) | FIB-4 > 1.3  and F sex  (N = 9) | FIB-4 < 1.3 and M sex  (N = 17) | FIB-4 > 1.3  and M sex  (N = 0) | p | p* |
| CIT [mcmol/l] | **47.4  (35.8–56.4)** | **30.4  (7.8–51.3)** | **41.2  (28.2–49.9)** | **-** | **.038** | .228 |
| I-FABP [pg/ml] | 1725.5 (1115.6–2289.8) | 2395.8 (1642.6–3042.9) | 1609.6 (1072.1–2405.8) | - | .085 | .510 |
| I-FABP/CIT ratio | **38.5  (21.5–61.8)** | **120.0  (46.7–299.1)** | **44.9  (33.3–64.7)** | **-** | **.036** | .216 |

**B) presence of the abdominal obesity**

| Analyzed parameter | ALT < ULN without AO  (N = 66) | ALT < ULN with AO  (N = 27) | ALT > ULN with AO  (N = 22) | p | p* |
| --- | --- | --- | --- | --- | --- |
| CIT [mcmol/l] | 46.2 (36.0–54.7) | 47.2 (28.8–55.7) | 44.0 (29.8–53.2) | .502 | .999 |
| I-FABP [pg/ml] | 1681.8 (1016.7–2364.4) | 2112.2 (1235.8–2405.8) | 1837.8 (1441.6–2384.6) | .535 | .999 |
| I-FABP/CIT ratio | 38.0 (19.1–58.8) | 45.0 (24.1–101.3) | 43.7 (32.1–62.8) | .208 | .832 |
| Analyzed parameter | ALT/AST < 1.33 without AO (N = 69) | ALT/AST < 1.33 with AO (N = 36) | ALT/AST ≥ 1.33 with AO (N = 10) | p | p* |
| CIT [mcmol/l] | 45.6 (35.9–54.6) | 46.3 (30.0–54.5) | 50.2 (37.4–57.2) | .542 | .999 |
| I-FABP [pg/ml] | 1725.5 (997.9–2413.0) | 2015.4 (1331.1–2385.6) | 1560.4 (1240.3–2222.0) | .655 | .999 |
| I-FABP/CIT ratio | 40.1 (20.7–62.0) | 43.6 (29.4–73.9) | 40.1 (20.7–51.7) | .422 | .999 |
| Analyzed parameter | HSI < 36.0 without AO  (N = 69) | HSI < 36.0 with AO (N = 15) | HSI > 36.0 with AO (N = 31) | p | p* |
| CIT [mcmol/l] | 45.6 (35.9–55.1) | 49.1 (31.5–55.7) | 45.3 (29.7–53.6) | .614 | .999 |
| I-FABP [pg/ml] | 1725.5 (1038.3–2413.0) | 1368.6 (1142.4–1780.3) | 2214.6 (1553.9–2412.5) | .148 | .592 |
| I-FABP/CIT ratio | 40.1 (20.7–62.0) | 35.1 (22.0–49.2) | 45.0 (36.5–76.8) | .213 | .852 |
| Analyzed parameter | FSI < -1.2 without AO (N = 70) | FSI < -1.2 with AO (N = 27) | FSI > -1.2 with AO (N = 18) | p | p* |
| CIT [mcmol/l] | 45.9 (35.9–54.7) | 47.8 (30.6–56.9) | 39.5 (29.8–51.1) | .372 | .999 |
| I-FABP [pg/ml] | 1723.3 (1016.7–2390.2) | 1918.6 (1262.4–2349.2) | 1972.2 (1441.6–2392.4) | .683 | .999 |
| I-FABP/CIT ratio | 39.5 (19.8–61.9) | 38.5 (24.1–76.8) | 45.8 (38.8–66.2) | .314 | .999 |
| Analyzed parameter | FIB-4 < 1.3 without AO  (N = 70) | FIB-4 < 1.3 with AO (N = 36) | FIB-4 > 1.3 with AO (N = 9) | p | p* |
| CIT [mcmol/l] | 46.2 (35.9–56.2) | 47.5 (31.5–55.6) | 30.4 (7.8–51.3) | .187 | .748 |
| I-FABP [pg/ml] | 1723.3 (956.3–2390.2) | 1724.3 (1331.1–2285.6) | 2395.8 (1642.6–3042.9) | .081 | .324 |
| I-FABP/CIT ratio | **39.5 (19.1–61.9)** | **41.6 (25.1–62.6)** | **120.0 (46.7–299.1)** | **.043** | .172 |

**C) MetS diagnosis**

| Analyzed parameter | ALT < ULN without MetS  (N = 75) | ALT > ULN without MetS  (N = 10) | | ALT < ULN and MetS  (N = 18) | | ALT > ULN and MetS  (N = 12) | p | p* |
| --- | --- | --- | --- | --- | --- | --- | --- | --- |
| CIT [mcmol/l] | 46.5  (35.9–56.6) | 49.4  (22.5–60.2) | | 37.2  (30.5–50.4) | | 39.1  (31.2–50.9) | .208 | .999 |
| I-FABP [pg/ml] | 1642.6 (1035.6–2335.4) | 2214.2 (1658.4–2847.7) | | 2154.0 (1381.3–2602.0) | | 1613.0 (1399.0–2256.3) | .434 | .999 |
| I-FABP/CIT ratio | 38.5  (18.8–61.8) | 44.9  (28.1–60.1) | | 47.9  (35.6–107.8) | | 43.7  (36.1–64.1) | .289 | .999 |
| Analyzed parameter | ALT/AST < 1.33 without MetS (N = 81) | | ALT/AST < 1.33 and MetS (N = 24) | | ALT/AST ≥ 1.33 and MetS (N = 10) | | p | p* |
| CIT [mcmol/l] | 46.0 (35.7–56.0) | | 37.2 (30.8–51.5) | | 50.2 (37.4–57.2) | | .310 | .999 |
| I-FABP [pg/ml] | 1725.5 (1038.3–2365.4) | | 2015.4 (1361.6–2404.1) | | 1560.4 (1240.3–2222.0) | | .655 | .999 |
| I-FABP/CIT ratio | 40.1 (20.7–62.5) | | 46.7 (34.1–68.2) | | 40.1 (20.7–51.7) | | .430 | .999 |
| Analyzed parameter | HSI < 36.0 without MetS  (N = 74) | HSI > 36.0 without MetS  (N = 11) | | HSI < 36.0 and MetS  (N = 10) | | HSI > 36.0 and MetS  (N = 30) | p | p* |
| CIT [mcmol/l] | **45.9  (35.8–56.0)** | **56.4  (49.4–59.0)** | | **48.9  (33.1–55.2)** | | **35.3  (26.2–49.7)** | **.021** | .126 |
| I-FABP [pg/ml] | 1681.8 (1039.6–2390.2) | 2214.6 (1179.9–2302.2) | | 1393.9 (1142.3–1827.5) | | 2210.3 (1585.0–2498.3) | .245 | .999 |
| I-FABP/CIT ratio | 39.5  (19.8–62.3) | 38.5  (19.2–49.4) | | 36.2  (23.9–46.4) | | 54.6  (42.1–124.3) | .064 | .384 |
| Analyzed parameter | FSI < -1.2 without MetS  (N = 83) | | FSI < -1.2 and MetS (N = 14) | | FSI > -1.2 and MetS (N = 18) | | p | p* |
| CIT [mcmol/l] | 46.3 (35.8–56.6) | | 42.7 (31.0–53.2) | | 39.5 (29.8–51.1) | | .238 | .952 |
| I-FABP [pg/ml] | 1725.5 (1040.9–2363.3) | | 1736.4 (1259.0–2585.5) | | 1972.2 (1441.6–2392.4) | | .768 | .999 |
| I-FABP/CIT ratio | 38.8 (20.0–62.2) | | 43.6 (27.3–68.9) | | 45.8 (38.8–66.2) | | .319 | .999 |
| Analyzed parameter | FIB-4 < 1.3 without MetS (N = 82) | | FIB-4 < 1.3 and MetS (N = 24) | | FIB-4 > 1.3 and MetS (N = 9) | | p | p* |
| CIT [mcmol/l] | **47.4 (36.0–57.1)** | | **37.2 (31.3–50.2)** | | **30.4 (7.8–51.3)** | | **.019** | .114 |
| I-FABP [pg/ml] | 1768.0 (1016.7–2364.4) | | 1613.0 (1361.6–2256.3) | | 2395.8 (1642.6–3042.9) | | .086 | .344 |
| I-FABP/CIT ratio | **38.7 (19.1–58.8)** | | **42.3 (34.1–64.1)** | | **120.0 (46.7–299.1)** | | **.026** | .104 |

**D) antidepressants intake**

No results available as the analysis was not powered enough.

**E) diagnosis of the specific mental disorder (depressive episode vs. mixed depressive and anxiety disorder)**

| Analyzed parameter | ALT < ULN and 6A70/6A71 (diagnosis according to ICD-11)  (N = 35) | ALT > ULN and 6A70/6A71 (diagnosis according to ICD-11)  (N = 7) | ALT < ULN and 6A72/6A73 (diagnosis according to ICD-11)  (N = 58) | ALT > ULN and 6A72/6A73 (diagnosis according to ICD-11)  (N = 15) | p | p* |
| --- | --- | --- | --- | --- | --- | --- |
| CIT [mcmol/l] | 47.2  (34.3–55.4) | 16.6  (6.0–38.8) | 46.2  (35.4–55.5) | 50.8  (37.8–55.0) | .192 | .999 |
| I-FABP [pg/ml],  mean (SD) | 1839.1 (1001.1) | 2030.2 (788.0) | 1806.3 (911.7) | 1871.8 (691.1) | .940 | .999 |
| I-FABP/CIT ratio | 42.1  (21.6–67.0) | 67.2  (43.1–486.1) | 38.2  (20.9–62.6) | 41.9  (28.4–50.3) | .255 | .999 |
| Analyzed parameter | HSI < 36.0 and 6A70/6A71 (diagnosis according to ICD-11)  (N = 37) | HSI > 36.0 and 6A70/6A71 (diagnosis according to ICD-11)  (N = 5) | HSI < 36.0 and 6A72/6A73 (diagnosis according to ICD-11)  (N = 60) | HSI > 36.0 and 6A72/6A73 (diagnosis according to ICD-11)  (N = 13) | p | p* |
| CIT [mcmol/l] | 45.9  (33.4–54.7) | 35.0  (17.4–50.1) | 46.3  (36.5–56.8) | 48.7  (31.2–53.7) | .512 | .999 |
| I-FABP [pg/ml], mean (SD) | 1736.2 (995.0) | 2364.9 (665.1) | 1789.1 (886.7) | 1889.3 (832.7) | .295 | .999 |
| I-FABP/CIT ratio | 40.1  (20.0–62.9) | 67.2  (42.1–137.1) | 37.2  (21.5–57.6) | 43.7  (32.1–62.9) | .163 | .999 |
| Analyzed parameter | FSI < -1.2 and 6A70/6A71 (diagnosis according to ICD-11)  (N = 33) | FSI > -1.2 and 6A70/6A71 (diagnosis according to ICD-11)  (N = 9) | FSI < -1.2 and 6A72/6A73 (diagnosis according to ICD-11)  (N = 51) | FSI > -1.2 and 6A72/6A73 (diagnosis according to ICD-11)  (N = 22) | p | p* |
| CIT [mcmol/l] | 45.9 (31.5–56.0) | 35.0 (17.4–42.6) | 46.5 (35.9–56.3) | 49.5 (32.0–52.1) | .267 | .999 |
| I-FABP [pg/ml], mean (SD) | 1856.9 (1016.3) | 1974.7 (438.5) | 1807.3 (867.3) | 1877.3 (891.1) | .974 | .999 |
| I-FABP/CIT ratio | 42.6 (23.3–71.1) | 67.2 (42.1–137.1) | 37.6 (21.8–62.1) | 44.9 (31.0–53.9) | .330 | .999 |

**F) supplements intake**

| Analyzed parameter | ALT < ULN without dietary supplements intake  (N = 49) | ALT > ULN without dietary supplements intake  (N = 8) | ALT < ULN with dietary supplements intake  (N = 44) | ALT > ULN with dietary supplements intake  (N = 14) | p | p* |
| --- | --- | --- | --- | --- | --- | --- |
| CIT [mcmol/l] | 47.8  (35.1–57.1) | 46.9  (38.9–52.5) | 44.0  (34.5–54.2) | 40.1  (8.7–55.0) | .587 | .999 |
| I-FABP [pg/ml] | 1560.8 (1039.6–1985.7) | 1613.0 (1468.5–2259.9) | 2209.0 (1315.8–2605.9) | 2028.6 (1463.0–2720.3) | .120 | .999 |
| I-FABP/CIT ratio | 36.9  (18.8–50.1) | 41.6  (36.1–43.5) | 47.5  (22.9–77.0) | 51.2  (28.1–369.9) | .124 | .999 |
| Analyzed parameter | HSI < 36.0 without dietary supplements intake  (N = 42) | HSI > 36.0 without dietary supplements intake  (N = 15) | HSI < 36.0 with dietary supplements intake  (N = 42) | HSI > 36.0 with dietary supplements intake  (N = 16) | p | p* |
| CIT [mcmol/l] | 47.3  (35.3–57.0) | 50.1  (36.0–53.6) | 44.1  (35.8–54.2) | 32.9  (7.8–52.2) | .388 | .999 |
| I-FABP [pg/ml] | **1554.2 (1105.0–1921.5)** | **1616.4 (1051.9–2317.3)** | **1843.4 (1200.9–2584.0)** | **2302.2 (2104.9–2739.7)** | **.030** | .180 |
| I-FABP/CIT ratio | **36.7  (20.0–49.3)** | **41.9  (23.4–45.9)** | **43.9  (21.5–71.1)** | **65.1  (43.3–323.2)** | **.024** | .144 |
| Analyzed parameter | FSI < -1.2 without dietary supplements intake  (N = 48) | FSI > -1.2 without dietary supplements intake  (N = 9) | FSI < -1.2 with dietary supplements intake  (N = 49) | FSI > -1.2 with dietary supplements intake  (N = 9) | p | p* |
| CIT [mcmol/l] | 47.8 (35.7–57.1) | 42.6 (35.6–51.3) | 44.1 (33.1–54.3) | 35.0 (7.8–50.8) | .321 | .999 |
| I-FABP [pg/ml] | 1567.4 (1072.9–2038.6) | 1609.6 (1340.6–2112.2) | 2145.5 (1282.4–2594.9) | 2351.0 (1832.1–2705.2) | .085 | .510 |
| I-FABP/CIT ratio | **37.0 (19.6–50.9)** | **41.9 (31.0–42.4)** | **45.7 (23.1–75.0)** | **63.0 (46.6–421.2)** | **.047** | .282 |

**G) smoking status**

| Analyzed parameter | ALT/AST  < 1.33 without smoking  (N = 87) | ALT/AST  ≥ 1.33 without smoking  (N = 10) | | ALT/AST  < 1.33 and  smoking  (N = 18) | | ALT/AST  ≥ 1.33 and  smoking  (N = 0) | p | p* |
| --- | --- | --- | --- | --- | --- | --- | --- | --- |
| CIT [mcmol/l] | 46.2  (34.3–55.1) | 50.2  (37.4–57.2) | | 45.3  (31.5–47.8) | | - | .754 | .999 |
| I-FABP [pg/ml],  mean (SD) | 1927.2 (924.2) | 1750.9  (813.3) | | 1444.9 (732.9) | | **-** | .123 | .738 |
| I-FABP/CIT ratio | 41.8  (23.9–66.2) | 40.1  (20.7–51.7) | | 37.0  (16.1–49.3) | | - | .447 | .999 |
| Analyzed parameter | HSI < 36.0 without smoking  (N = 72) | HSI > 36.0 without smoking  (N = 25) | | HSI < 36.0 and  smoking  (N = 12) | | HSI > 36.0 and  smoking  (N = 6) | p | p* |
| CIT [mcmol/l] | 47.3  (35.3–57.0) | 50.1  (36.0–53.6) | | 44.1  (35.8–54.2) | | 32.9  (7.8–52.2) | .388 | .999 |
| I-FABP [pg/ml] | **1810.5 (1175.0–2463.1)** | **2214.6 (1511.2–2429.3)** | | **1222.5 (729.8–1598.4)** | | **2224.0 (1746.5–2264.8)** | **.025** | .150 |
| I-FABP/CIT ratio | 40.1  (22.8–62.9) | 44.9  (37.8–67.2) | | 25.8  (14.7–38.9) | | 66.8  (42.0–79.7) | .074 | .444 |
| Analyzed parameter | FSI < -1.2 without smoking (N = 79) | | FSI > -1.2 without smoking (N = 18) | | FSI < -1.2 and smoking  (N = 18) | | p | p* |
| CIT [mcmol/l] | 47.2 (35.6–56.5) | | 39.5 (29.8–51.1) | | 45.3 (31.5–47.8) | | .271 | .999 |
| I-FABP [pg/ml] | 1909.8 (943.2) | | 1904.4 (779.6) | | 1444.9 (732.9) | | .146 | .584 |
| I-FABP/CIT ratio | 40.6 (23.1–62.9) | | 45.8 (38.8–66.2) | | 37.0 (16.1–49.3) | | .253 | .999 |

**H) comorbidities**

| Analyzed parameter | ALT < ULN without comorbidities  (N = 47) | ALT > ULN without comorbidities  (N = 7) | | ALT < ULN and comorbidities  (N = 46) | | ALT > ULN and comorbidities  (N = 15) | p | p* |
| --- | --- | --- | --- | --- | --- | --- | --- | --- |
| CIT [mcmol/l] | 47.3  (35.2–55.7) | 40.0  (25.8–56.8) | | 46.3  (34.0–55.3) | | 45.3  (30.5–52.4) | .864 | .999 |
| I-FABP [pg/ml] | 1631.6 (1105.0–2335.4) | 1616.4 (1112.6–2282.4) | | 1900.9 (1131.9–2451.2) | | 1843.4 (1553.9–2619.6) | .663 | .999 |
| I-FABP/CIT ratio | 38.5  (21.5–54.6) | 48.5  (28.1–64.7) | | 42.5  (20.3–72.5) | | 42.4  (36.5–58.5) | .566 | .999 |
| Analyzed parameter | HSI < 36.0 without comorbidities  (N = 46) | HSI > 36.0 without comorbidities  (N = 8) | | HSI < 36.0 and comorbidities  (N = 38) | | HSI > 36.0 and comorbidities  (N = 23) | p | p* |
| CIT [mcmol/l] | 44.0  (34.0–54.3) | 55.0  (46.3–58.7) | | 46.9  (42.3–56.6) | | 36.4  (28.2–51.0) | .138 | .828 |
| I-FABP [pg/ml] | 1614.9 (992.8–2308.2) | 2163.0 (1566.9–2339.3) | | 1768.0 (1184.9–2434.7) | | 2224.8 (1553.9–2569.0) | .384 | .999 |
| I-FABP/CIT ratio | 38.4  (21.1–62.3) | 40.3  (29.6–51.8) | | 35.6  (21.5–57.7) | | 46.7  (39.6–101.3) | .304 | .999 |
| Analyzed parameter | FSI < -1.2 without comorbidities (N = 49) | | FSI < -1.2 and comorbidities (N = 48) | | FSI > -1.2 and comorbidities (N = 18) | | p | p* |
| CIT [mcmol/l] | 44.8 (34.9–56.4) | | 46.5 (35.2–55.7) | | 39.5 (29.8–51.1) | | .375 | .999 |
| I-FABP [pg/ml] | 1616.5 (949.9–2312.6) | | 1900.9 (1199.9–2524.9) | | 1972.2 (1441.6–2392.4) | | .455 | .999 |
| I-FABP/CIT ratio | 38.0 (20.7–58.4) | | 41.2 (24.4–71.6) | | 45.8 (38.8–66.2) | | .248 | .992 |

Abbreviations: 6A70 = depressive episode; 6A71 = recurrent depression; 6A72 = dysthymia; 6A73 – mixed depressive and anxiety disorder; ALT = alanine transaminase; AO = abdominal obesity; APRI = AST-to-platelet ratio index; CIT = citrulline; F = female sex; FIB-4 = fibrosis-4 index; FSI = Framingham Steatosis Index; HSI = hepatic steatosis index, I-FABP = intestinal fatty acid binding protein; M = male sex; ULN = upper limit of normal; p*—the p-value after Bonferroni correction.
